# Wide diversity of fungal species found in wellwater for human consumption: an analytical cross-sectional study

**DOI:** 10.1590/1516-3180.2019.0313160919

**Published:** 2020-03-06

**Authors:** Máira Gazzola Arroyo, Oleci Pereira Frota, Jacqueline Tanury Macruz Peresi, Natalia Seron Brizzotti-Mazuchi, Adriano Menis Ferreira, Marcelo Alessandro Rigotti, Alvaro Francisco Lopes de Sousa, Denise de Andrade, Elza Maria Castilho, Margarete Teresa Gottardo de Almeida

**Affiliations:** I MSc. Microbiologist, Postgraduate Program on Microbiology, Universidade Estadual Paulista (UNESP), São José do Rio Preto (SP), Brazil.; II RN, PhD. Adjunct Research Professor, Postgraduate Program on Nursing, Universidade Federal do Mato Grosso do Sul (UFMS), Campo Grande (MS), Brazil.; III MSc. Pharmacist and Scientific Researcher, Adolfo Lutz Institute, Regional Laboratory of São José do Rio Preto, São José do Rio Preto (SP), Brazil.; IV MSc. Biologist, Department of Infectious and Parasitic Diseases, Faculdade de Medicina de São José do Rio Preto (FAMERP), São José do Rio Preto (SP), Brazil.; V RN, PhD. Associate Professor, Postgraduate Programs on Nursing and Medicine, Universidade Federal do Mato Grosso do Sul (UFMS), Três Lagoas (MS), Brazil.; VI RN, PhD. Professor, Undergraduate Nursing Course, Universidade Federal do Mato Grosso do Sul (UFMS), Três Lagoas (MS), Brazil.; VII RN. Doctoral Student, Department of General and Specialized Nursing, Escola de Enfermagem de Ribeirão Preto da Universidade de São Paulo (EERP-USP), Ribeirão Preto (SP), Brazil; and Doctoral Student, Institute of Hygiene and Medicine Tropical, New University of Lisbon, Portugal.; VIII RN, PhD. Associate Professor, Department of General and Specialized Nursing, Escola de Enfermagem de Ribeirão Preto da Universidade de São Paulo (EERP-USP), Ribeirão Preto (SP), Brazil.; IX PhD. Biologist and Assistant Professor, Department of Molecular Biology, School of Medicine of São José do Rio Preto, São José do Rio Preto (SP), Brazil.; X PhD. Microbiologist and Assistant Professor, Department of Infectious and Parasitic Diseases, School of Medicine of São José do Rio Preto, São José do Rio Preto (SP), Brazil.

**Keywords:** Water quality, Water wells, Fungi, Chlorine, pH, Free residual chlorine, Microorganisms

## Abstract

**BACKGROUND:**

Fungi are ubiquitous in the environment. They are able to grow in water and many of them may be opportunistic pathogens.

**OBJECTIVE:**

The aims were to identify fungi in registered wells (RWs) and nonregistered wells (NRWs) that tap into groundwater; and to correlate the results from physicochemical assays on this water (free residual chlorine and pH) with the presence of fungi.

**DATA AND SETTING:**

Analytical cross-sectional quantitative study on groundwater wells in São José do Rio Preto, São Paulo, Brazil.

**METHODS:**

52 samples of 500 ml of water were collected from RWs and 107 from NRWs. These were sent to a microbiology laboratory to identify any fungi that were present. In addition, free residual chlorine and pH were measured immediately after sample collection. Several statistical analysis tests were used.

**RESULTS:**

Fungal contamination was present in 78.8% of the samples from RWs and 81.3% from NRWs. Filamentous fungi were more prevalent than yeast in both types of wells. There was no significant difference in presence of fungi according to whether chloride and pH were within recommended levels in RWs; or according to whether pH was within recommended levels in NRWs. Furthermore, there was no statistical difference in the levels of fungal contamination between RWs and NRWs.

**CONCLUSION:**

Both RWs and NRWs are potential reservoirs for many types of fungi. Many of these may become opportunistic pathogens if they infect immunosuppressed individuals. Furthermore, this study confirms that fungi are able to grow even when chlorine and pH parameters are within the standards recommended.

## INTRODUCTION

Ensuring human health is directly related to water quality. The water supply needs to meet microbiological, physical and chemical standards, for it to be in an optimal condition for consumption.^1^

Underground water is one of the various types of drinking water sources. Although it is considered safe, given the filtration power of the soil, contamination with external microbes may be present. This event is common when water collection wells are constructed irregularly, thus favoring the entry of microorganisms and representing a risk to the health of the population.^2^^,^^3^

In the municipality of the present study, the municipal health surveillance agency is responsible for enforcing the quality of underground water from wells. All wells drilled need to be registered in the health surveillance system for monitoring to verify water quality.

However, irregular settlements exist within this municipality, which became inhabited by their populations at a time before the mandatory documentation came into existence. Thus, there is no legal basis for provision of a municipal water supply to these settlements. For this reason, wells have been drilled to obtain local water supplies. But because these are irregular settlements, the wells drilled there are also irregular, i.e. they are not registered in the health surveillance system. As a consequence of these irregularities, the water quality of these places is not monitored by the agency that should be responsible for this. This endangers the health of the local population, given that its water supply is left susceptible to contamination by microorganisms.

Within this context, it is known that fungi are ubiquitous in the environment. The fact that many of them may be opportunistic pathogens, causing many types of health problems, including allergies, mycosis and presence of biofilm and mycotoxins, is a subject of growing interest.^4^^,^^5^

Although the evidence is scarce, some studies have proven that correlations exist between fungi found in hospitalized patients and the same fungi found in the water from taps (faucets) and showers in hospitals.^6^^,^^7^

Aquatic environments favor the survival of fungi, because under these ideal nutrient and temperature conditions, fungi are capable of reproducing.^8^ Consequently, water supply networks, reservoirs, tanks, faucets and showerheads may harbor different species, on inanimate surfaces. Thus, such environments are potential disease-transmitting vehicles.^6^

In addition to these parameters, it is also important that the quantity of chlorine in water should be monitored, since chlorine is the most effective agent against the growth of microorganisms.^1^

Therefore, research evaluating the occurrence and distribution of fungi in drinking water is necessary, since contamination by these agents may be harmful to health, especially among the most vulnerable individuals.

## OBJECTIVE

The objectives of this study were to identify the presence of fungi in wells that tap into groundwater, i.e. both registered wells that are within the local health surveillance system and nonregistered wells in irregular or illegal settlements (unsupervised wells outside of the health surveillance system); and to correlate the results from physicochemical assays on this water (free residual chlorine and pH) with the presence of fungi.

## METHODS

### Study design, period, setting

This analytical cross-sectional study was conducted in São José do Rio Preto, São Paulo, Brazil, from September 2011 to June 2012.

The locations of registered wells were ascertained through consulting the list of these wells that is held in the health surveillance system. In relation to irregularly drilled wells, i.e. nonregistered wells, a list with addresses of the irregular settlements was passed on from the municipal authorities to the health surveillance professionals so that water could be collected from these places.

One 500 ml sample was collected per well: 52 from registered wells and 107 from nonregistered wells (a greater number of nonregistered wells was included in the study because there were more of them in the municipality).

### Sample collection procedures

The water samples collected came from taps that provided a water supply from their respective well. Before the sample was collected, each tap was disinfected with 70% alcohol and then the water was allowed to flow continuously for two minutes. Following this, 500 ml were collected in a sterile container containing 1.8% sodium thiosulfate. These containers were then transported in isothermal boxes to a microbiology laboratory for mycological analysis.^9^

### Sample analysis

For sample analysis, the membrane filter method was used. This method stands out as the most viable laboratory procedure for counting microorganism colonies in water. The protocol for this method is described in the “*Standard Methods for the Examination of Water Protocol and Wastewater”.*^9^

The whole volume of water that had been collected was filtered through sterile cellulose membranes (47 mm; 0.45 µm). These filters have the capacity to retain various types of microorganisms such as fungi that are isolated from water.

The membranes were then placed on the surface of Petri dishes containing Sabouraud dextrose (containing chloramphenicol) for culturing, with incubation for 15 days at 30 °C. After this period, all colonies of morphologically distinct fungi were selected; if identical, only one isolate was considered.^10^^,^^11^

The filamentous fungi were identified through their macroscopic and microscopic features and through microculturing to stimulate development of fruiting bodies. For this, a small piece of Sabouraud dextrose agar was placed on a sterilized slide lying in a sterilized Petri dish. Fungi from a recent subculture were grown on all four sides of the agar block, which was covered with a sterilized coverslip. Next, 2 ml of sterile distilled water were added to the dish to prevent desiccation of the growth medium. A top was placed on the dish and its contents were left to incubate at 25 °C for seven to ten days, until development of hyphae was detected. After this fungal growth, the coverslip was removed using tweezers and a drop of lactophenol cotton blue stain was added to the material in order to view spores and hyphae using an optical microscope.^10^

In order to identify any yeasts that were isolated from the water, physiological tests consisting of assimilation of different carbohydrates (maltose, sucrose, lactose, galactose, melibiose, cellobiose, dextrose, inositol, xylose, raffinose, trehalose and dulcitol) and nitrogen sources (peptone and potassium nitrate) were performed. These tests were done using the pour-plate technique: 2 ml of a suspension of each yeast were homogenized in each mixed medium (yeast nitrogen base and yeast carbon base) and were distributed into two Petri dishes for culturing.

Small aliquots of each different carbohydrate were added to the surface of the carbon-free medium to serve as carbon sources. In another dish, nitrogen sources were placed on the surface of the nitrogen-free medium. After incubation at 30 °C for 24 to 48 hours, the results were read.^11^

### Physical and chemical analysis

The water-free residual chlorine content and pH were measured immediately after the water sample collection, by professionals from the local health surveillance system. To determine the free residual chlorine content, 10 ml of each sample and 0.5 ml of *N, N*-diethyl-*p*-paraphenylenediamine reactant (DPD) were added to a cuvette. The mixture was stirred to ensure homogeneity, and measurement was done using an electronic colorimeter device (HI96711C; Hanna Instruments). For the free residual chlorine levels to be within the range recommended by the World Health Organization (WHO),^1^ the concentrations needed to be between 0.2 mg/l and 2 mg/l. pH was determined with the aid of a benchtop pH meter (PH250; Policontrol), which had previously been calibrated using standard solutions. The electrode of the device was immersed in an aliquot from each sample. The recommended water pH range was 6.0 to 9.5.^1^

### Statistical analysis

The association between presence of fungi and free residual chlorine levels and pH parameters within the recommendations (for both wells) was investigated using the Mann-Whitney nonparametric test (which compares the medians of independent groups). The G-test (used to evaluate whether variables are associated or not) was used to investigate whether there was any association between the status of the well (registered or nonregistered) and the presence of fungi. The significance level was set at P ≤ 0.05.

## RESULTS

Most of the samples showed fungal contamination: 80% (128/159) overall; 78.8% (41/52) for registered wells; and 81.3% (87/107) for nonregistered wells. A wide variety of fungi were observed, regardless of the source of the water (Table 1). In registered wells, 75% (39/52) and 7.7% (4/52) of the samples were positive for filamentous fungi and yeast, respectively. In nonregistered wells, filamentous fungi were recovered from 74.7% (80/107) of the wells and yeast in 25.2% (27/107).

**Table 1.** Distribution of fungal isolates from registered and nonregistered wells

Registered wells (RWs)				Nonregistered wells (NRWs)			
Filamentous	N	Yeasts	n	Filamentous	n	Yeasts	n
*Aspergillus fumigatus*	28	*Candida guilliermondii*	3	*Aspergillus fumigatus*	21	*Aureobasidium pullulans*	7
*Acremonium hyalinulum*	4	*Candida parapsilosis*	1	*Penicillium commune*	19	*Candida guilliermondii*	5
*Aspergillus penicillioides*	3	*Blastoschizomyces capitatus*	1	*Penicillium decumbens*	8	*Candida intermedia*	4
*Aspergillus japonicus*	2	*Aureobasidium pullulans*	1	*Penicillium expansum*	6	*Trichosporon asahii*	4
*Penicillium marneffei*	2			*Fusarium incarnatum*	6	*Candida tropicalis*	3
*Curvularia clavata*	1			*Aspergillus penicillioides*	5	*Candida glabrata*	2
*Fusarium incarnatum*	1			*Basidiobolus ranarum*	5	*Candida lusitaniae*	2
*Penicillium marquandii*	1			*Penicillium citrinum*	4	*Candida parapsilosis*	2
*Penicillium expansum*	1			*Penicillium spinulosum*	4	*Trichosporon* *mucoides*	2
*Aspergillus caesiellus*	1			*Aspergillus japonicus*	4	*Candida famata*	2
*Aspergillus flavus*	1			*Acremonium hyalinulum*	4	*Candida silvicola*	1
				*Penicillium purpurogenum*	3	*Kodamaea ohmeri*	1
				*Aspergillus flavus*	3	*Rhodotorula minuta*	1
				*Scytalidium hyalinum*	3	*Rhodotorula glutinis*	1
				*Curvularia clavata*	2	*Zygosaccharomyces florentinus*	1
				*Aspergillus clavatus*	2		
				*Curvularia clavata*	1		
				Other genera/species*	16		
Total	45	Total	6	Total	116	Total	38

n = total number of isolates.

*Fusarium solani, *Acremonium potronii*, *Alternaria alternata*, *Mucor racemosus*, *Fusarium sacchari*, *Fusarium hyalinum*, *Penicillium marquandii*, *Absidia cylindrospora*, *Scedosporium dehoogii*, *Scedosporium apiospermum*, *Trichoderma harzianum*, *Penicillium lilacinus*, *Penicillium verruculosum*, *Aspergillus versicolor*, *Aspergillus candidus*, *Aspergillus caesiellus*.

*Aspergillus fumigatus* was most prevalent in both types of wells, followed by *Penicillium commune* in the nonregistered wells. Regarding yeasts, *Candida guilliermondii* was the species most found in registered wells and the second in nonregistered wells, while *Aureobasidium pullulans* was the species most found in nonregistered wells.

Table 2 shows the numbers of samples with presence of fungi, correlated with the chloride and pH levels in both types of well.

**Table 2.** Numbers of samples with presence of fungi, correlated with chloride and pH levels

Sampled	Chlorine (0.2 to 2.0 mg/l)		pH 6.0 to 9.5	
	RW	NRW	RW	NRW
Within recommended levels	20	0	40	87
Exceeded recommended levels	21	87	1	0
Total	41	87	41	87

RW = registered well; NRW = nonregistered well.

The statistical results indicated that there was no significant difference in presence of fungi according to whether chloride levels (P = 0.3804) and pH levels (P = 0.3187) within the recommendations, in registered wells. Similarly, there was no statistical difference in presence of fungi according to whether pH levels (P = 0.1396) were within the recommendations, in nonregistered wells.

Furthermore, no statistical difference regarding the presence of fungi was observed between registered and nonregistered wells (P = 0.7146).

## DISCUSSION

The diversity of fungi in drinking water has been documented and is a concern for researchers, scientists, institutions and organizations around the world. However, the scarcity of large-scale studies and the lack of uniformity of the available research make it difficult to have an exact notion of the complexity of this phenomenon worldwide.^4^^,^^5^ It is known that the fungi *Aspergillus* spp. and *Penicillium* spp. have greater adaptation to aquatic environments, and that they commonly have the ability to survive in treated water. This was seen in the present study, in which both of these genera had high survival rates.^4^^,^^6^

The most common species in both wells was *Aspergillus fumigatus*. Presence of this fungus may expose the human or animal host to the risk of diseases: pneumonia, systemic diseases, acute or chronic infections and allergies, especially in vulnerable individuals.^12^^,^^13^

In addition, special attention should be given to the large number of *Penicillium commune* isolates in the present study. Kadaifciler and Demirel reported that *Penicillium* spp. were the predominant group in the water system, with the capacity to produce mycotoxins.^5^ Among immunocompromised patients, this fungus may lead to severe diseases, especially those that are acquired through inhalation, even if it is only present in small amounts.^4^^,^^5^

Although yeast species were less commonly found in the present investigation, their diversity is corroborated by data from other studies, involving water from different sources.^14^ Like filamentous fungi, yeasts can also cause various kinds of diseases. After ingestion of water containing yeast, this yeast reaches the bloodstream through translocation, and then reaches other organs. Yeast also gives rise to a risk of infection when in contact with open wounds.^14^^,^^15^

Similarly, *Aureobasidium pullulans*, which was detected in water from both types of wells (Table 1), poses a risk to the health of immunosuppressed consumers, because this species can survival in water distribution systems.^16^*Candida guilliermondii* was commonly found concomitantly in the present study, regardless of the origin, which may indicate a potential risk to health. Presence of this species in the water of dental care units, in another study.^14^

Chlorine is the most common sanitizing agent used in water to eliminate disease-causing bacteria.^1^ However, it did not have the same effect on fungi, since fungi were recovered from samples that had been correctly chlorinated. The present study showed that *A. fumigatus* was the most frequently found filamentous fungus in both types of wells. This prevalence may also have occurred because of its resistance to chlorine, as demonstrated in a previous study, in which survival of this species in chlorinated waters was reported.^17^

Another study showed that *Aspergillus* spp. was also able to grow in treated water, and demonstrated the occurrence of chlorine-resistant *Candida* isolates.^18^ This was also observed in the present study, especially in relation to *A. pullulans* and *C guilliermondii*, which are yeasts with greater numbers of isolates and probable resistance to chlorine.

Moreover, no association was observed between fungal growth and chloride parameters (P = 0.3804), indicating that the fungi were capable of developing in both chlorinated and non-chlorinated water (Table 2).

Most microorganisms multiply better within a particular pH range, called the optimal range, and this differs between species. There are fungi that have the ability to grow in media ranging from basic to acidic. Thus, if their nutritional requirements are met, it is likely that fungi will develop in different types of environments.^19^ This was observed in the present study, in which there was high incidence of fungi in most of the samples in which the pH was within the recommended range (Table 2).

Even though there was no statistical difference in the presence of fungi in water with the recommended pH parameters (for both types of wells), growth of these microorganisms under the physical conditions studied was demonstrated.

We did not observe any statistical difference in fungal growth (P = 0.7146) between the registered and nonregistered wells (Table 2), even though it had been expected that nonregistered wells (which are unsupervised) would have had higher levels of contamination. This observation strengthens the importance of the present study, which showed contamination even in the registered wells. The fungi isolated from both types of wells may cause disease, especially among immunocompromised patients. Thus, these findings have an important impact on public health and emphasize the need to monitor these environments.

## CONCLUSIONS

Both registered wells and nonregistered wells were shown to be potential reservoirs of many types of fungi, including filamentous fungi and yeast. Many of these may become opportunistic pathogens when they infect immunosuppressed individuals. Furthermore, it was confirmed in this study that fungi were able to grow even when the chlorine and pH levels were within the standards recommended.
